# The GAS PefCD exporter is a MDR system that confers resistance to heme and structurally diverse compounds

**DOI:** 10.1186/s12866-016-0687-6

**Published:** 2016-04-19

**Authors:** Ankita J. Sachla, Zehava Eichenbaum

**Affiliations:** Department of Biology, College of Arts and Sciences, Georgia State University, P.O. Box 4010, Atlanta, GA 30302-4010 USA

**Keywords:** Mutational analysis, Multi-drug exporter, Doxorubicin, Gram-positive, PefRCD, DNA damage detection, Heme content

## Abstract

**Background:**

Group A streptococcus (GAS) is the etiological agent of a variety of local and invasive infections as well as post-infection complications in humans. This β-hemolytic bacterium encounters environmental heme in vivo in a concentration that depends on the infection type and stage. While heme is a noxious molecule, the regulation of cellular heme levels and toxicity is underappreciated in GAS. We previously reported that heme induces three GAS genes that are similar to the *pefRCD* (*porphyrin regulated efflux*) genes from group B streptococcus. Here, we investigate the contributions of the GAS *pef* genes to heme management and physiology.

**Results:**

*In silico* analysis revealed that the PefCD proteins entail a Class-1 ABC-type transporter with homology to selected MDR systems from Gram-positive bacteria. RT-PCR experiments confirmed that the *pefRCD* genes are transcribed to polycistronic mRNA and that a *pefC* insertion inactivation mutant lost the expression of both *pefC* and *pefD genes*. This mutant was hypersensitive to heme, exhibiting significant growth inhibition already in the presence of 1 μM heme. In addition, the *pefC* mutant was more sensitive to several drugs and nucleic acid dyes and demonstrated higher cellular accumulation of heme in comparison with the wild type and the complemented strains. Finally, the absence of the PefCD transporter potentiated the damaging effects of heme on GAS building blocks including lipids and DNA.

**Conclusion:**

We show here that in GAS, the *pefCD* genes encode a multi-drug efflux system that allows the bacterium to circumvent the challenges imposed by labile heme. This is the first heme resistance machinery described in GAS.

## Background

*Streptococcus pyogenes* or Group A Streptococcus (GAS) is a Gram positive, β- hemolytic, human pathogen transmitted *via* respiratory droplets and direct contact. GAS is responsible for a diverse spectrum of diseases ranging from superficial (e.g., pharyngitis, impetigo and pyoderma) to severe invasive infections and systemic manifestations (such as necrotizing fasciitis and streptococcal toxic shock syndrome). In addition, simple GAS infections can trigger autoimmune reactions in some patients leading to neurological disorders, glomerulonephritis, or acute rheumatic fever [1, 2]. GAS encodes a large collection of virulence factors, which act to promote infections and pathologies by various means such as bacterial adherence and invasion, evasion of the host immune surveillance and nutrient acquisition [3]. Due to the high morbidity associated with GAS related illnesses, this pathogen is ranked 9th among the world’s leading infectious agents [4]. Recent global estimates suggest that every year about 600 million GAS infections occur, accounting for ~ 600,000 deaths [5]. The reported increase in antibiotic resistance, emergence of new strains, and absence of vaccine programs suggest that the disease burden inflicted by GAS is likely to rise [6, 7].

GAS requires iron for growth and can retrieve the metal from heme [8]. The cytolysins produce by GAS provide the pathogen with access to the host intracellular pool of hemoproteins. GAS proceeds with heme uptake using the streptococcal iron acquisition (*sia*) operon: heme is extracted and captured on the bacterial surface by the Streptococcal hemoproteins receptor (Shr), relayed to the streptococcal hemoprotein (Shp), and subsequently to the substrate binding protein, SiaA (HtsA). The SiaBC importer then internalizes the heme [9–13]. Although heme is nutritionally beneficial, its pro-oxidant nature renders it hazardous in surplus quantities [14]. We recently observed that exposure to sub-lethal heme concentrations elicits a global stress response in GAS [15]. This observation underscores the harmful potential of heme. Heme toxicity seems particularly relevant to the patho-physiology of the β-hemolytic pathogen, and could impose as a significant challenge for GAS growth during invasive infections.

Heme-mediated cell injuries are primarily accredited to the coordinated iron element. The dynamic existence of iron in two redox and spin states within heme allows it to react with multiple biological entities including proteins, nucleic acids and lipids. This effect is exacerbated by the participation of iron in Fenton reactions, which lead to generation of radicals or reactive oxygen species [16]. In addition to iron toxicity, the lipophilic nature of the protoporphyrin-ring (PPIX) is associated with photosensitivity and insertion of heme into membranes. This process was shown to undermine membrane permeability and further potentiates the release of free-heme from erythrocytes [17, 18]. In mammalians, increased and persistent flux of free-heme in the vascular system is associated with significant inflammation and pathology [19].

To manage heme toxicity, bacteria carefully control heme uptake according to cellular demands. In GAS for example, a metalloregulatory protein named MtsR, directly represses the expression of the *sia* operon in the presence of iron [20]. In addition to limiting heme uptake, bacteria employ various sequestration, degradation, and active efflux mechanisms. The export of heme excess and its role in protection against toxicity has been increasingly recognized in bacteria. The multiple-transferable-resistance (MtrCDE) efflux system from *Neisseria gonorrhea* which, expels hydrophobic antibacterial agents [21], also facilitate resistance to PPIX, heme, and other porphyrin-based compounds. Inactivation of the *mtrCDE* pump resulted in increased gonococci sensitivity to porphyrins and metalloporphyrins, while overexpression of this system endowed cells with an increased tolerance to porphyrin based compounds [22]. The *E. coli* MacAB pump is another example of an active exporter with broad specificity that contributes to heme tolerance. MacAB (with the TolC outer membrane channel protein) enables eneterotoxin secretion and confers resistance to macrolides [23, 24]. In addition, MacAB-TolC serves as the major PPIX exporter in *E. coli* [25].

In Gram-positive organisms, the archetype of an exporter that mediates heme tolerance in several bacteria including *Bacillus anthracis* and *Lactococcus lactis* was identified first in *Staphylococcus aureus* and named heme-regulated transporter (HrtAB) [26–29]. Work performed in *L. lactis* suggested that this system exports heme directly from the membrane, acting to limit heme accumulation in this compartment [29, 30]. The expression of the *hrtAB* genes is tightly regulated according to heme availability *via* the two-component system HssRS (in *S. aureus* and *B. anthracis*) or the heme-dependent repressor, HrtR (*L. lactis*) [28, 29, 31]. The presence of the HrtAB transporter and the associated regulatory network bestows bacteria with elevated heme sensing and resistance capacity. Studies in a mouse infection model demonstrated that the *HrtAB* promoter in *B. anthracis* is induced *in vivo*, suggesting that the HrtAB transporter is recruited during infection, presumably to alleviate heme toxicity [28]. Interestingly, while both species exhibit similar sensitivity to environmental heme, the loss of *hrtA* in *B. anthracis* has a more pronounced impact than in *S. aureus*, implying that heme sensing and/or detoxification by the HssRS and HrtAB systems are more effective in *B. anthracis* than in *S. aureus*. [28]. The genome of Group B streptococcus (GBS) contains putative *hrtAB* homologs that are regulated by heme availability. However, contribution of these genes to heme efflux and tolerance in GBS has yet to be determined. In addition, GBS carries a regulon called *porphyrin regulated efflux (pef)* whose expression is controlled by PefR, a MarR-like repressor [32]. Heme or PPIX allow for transcriptional activation of the *pef* regulon by relieving PefR binding to its operator. Th*e pef* regulon consists of at least two separate gene clusters (*pefAB* and *peRCD*) that encode two distinct transporters, *pefAB* and *pefCD*. Inactivation of the GBS *pef* transport system results in increased sensitivity to heme and intracellular buildup of heme and PPIX. Overexpression of these systems led to heme depletion and a defect in both respiratory metabolism and virulence [32]. In *Listeria monocytogenes,* an ATP-dependent transporter, FrvA (Fur regulated virulence factor A), was implicated in heme export and protection from heme toxicity [33]. Notably, the FrvA transporter is essential for the systemic infections in mice models.

Mechanisms of heme tolerance are poorly understood in GAS. We recently identified a three-gene cluster in GAS that shares significant similarity to the GBS *pefRCD* genes. We demonstrated that these genes are up regulated in response to heme exposure and implicated the PefR homolog in heme sensing and regulation of the gene cluster [15]. In this study, we set to determine the function of the *pefCD* genes in GAS physiology.

## Results

### The pefCD genes encode a conserved Class-1 ABC exporter

**Fig. 1:**
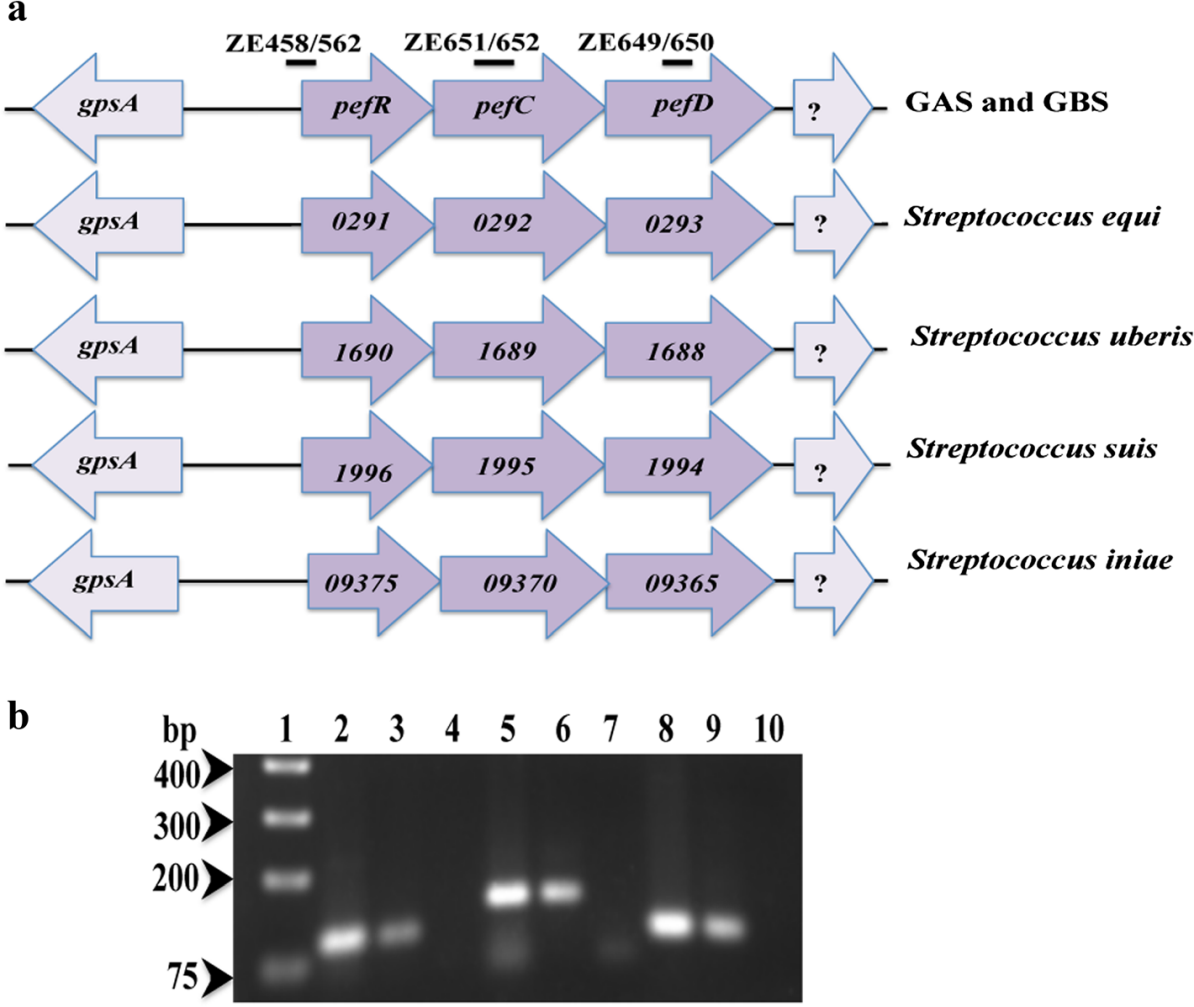
The *pef*RCD genes entail an operon prevalent in human and zoonotic streptococci. **a** The genomic arrangement and occurrence of the *pefRCD* genes in GAS (MGAS5005 strain) and GBS (NEM316 strain) is also conserved among members of the pyogenic cluster namely, *S. equi* (4047 strain), *S. uberis* (0140 J strain), and *S. iniae* (ISET0901). Additionally, this system is found in *S. suis* (JS14 strain). These PefCD homologs are referred to as SatAB in the literature [37]. The *pef* operon consists of a MarR-like transcriptional regulator (locus tag for strains tested: *Spy_0195, gbs1402, SEQ_0290, SUB_1690, SSUJS14_1996,* and *DQ08_09375*) controlling an adjacent ABC-type efflux system (*Spy_0196-97, gbs1401-00, SEQ_0292-93, SUB_1689-88, SSUJS14_1995-94,* and *DQ08_09370-65*). The neighboring genes upstream and downstream from the *pef* operon consist of lipid metabolism (*gpsA*) and unknown function, respectively. **b** The GAS *pefRCD* genes are co-transcribed. RT-PCR analysis was preformed with 0.8 μg RNA extracted from NZ131 strain. cDNA synthesized by use of the *pefD* antisense primer (ZE650), was amplified by PCR with gene specific primers and fractionated on 1.2 % agarose gel (lanes 2, 5, and 8). PCR was also conducted using genomic DNA (lanes 3, 6, and 9) or RNA (without RT reaction, lanes 4, 7, and 10) as templates. Fragments from the following genes were amplified: *pefR,* lanes 2, 3 and 4; *pefC,* lanes 5, 6 and 7; and *pefD*, lanes 8, 9, and 10. The lines above the gene diagram in Figure-1A depict the amplified regions and the primer sets used in the PCR are included on top. The sizes of molecular mass standards are indicated to the left of the gel

We recently reported the transcriptional activation of a 3-gene cluster (MGAS5005 spy_0195, 0196, and 0197) ensuing GAS exposure to environmental heme. We named these GAS genes as *pefRCD* based on similarity in genetic organization, primary sequence, and regulation to the previously described *pefRCD* operon in GBS [15, 32] (Fig. 1a). Our analysis here showed that the *pefRCD* genes are highly conserved among GAS strains exhibiting 100 % identity for PefR, 98 % identity for PefC, and 99 % identity for PefD protein (for all 20 strains in the database). The *pefCD* genes are annotated in GAS genome as the subunits of a putative multi-drug resistance (MDR) system. Sequence examination indicated that the *pefCD* genes code for the two subunits of a heteroligomeric ATP-dependent exporter. Both ORFs contain, at the N terminus, an integral membrane (IM) permease domain from the superfamily of ATP binding cassettes fused to an ATP-hydrolyzing domain (ABC, also referred to as the nucleotide binding domain). This domain organization is of the Class-1 ABC-type transporter family [34]. This transporter class is comprised of systems with fused IM and ABC domains and contains the vast majority of ABC-type export systems [34]. Our recent BLAST analysis against the transporter classification (TC) database (http://www.tcdb.org) [35] revealed that the PefCD proteins consists of a transporter from the Drug Exporter-4 sub class (3.A.1.135). Importantly, this analysis revealed that PefCD exporter is identical to a previously described transporter from the GAS strain JRS4, named Rsc (regulated by stress and Cov) after its regulation by the response regulator CovR [36]. The substrate (s) of the Rsc system was not identified but the transporter was found to be required for bacterial growth at high temperature. The nearest relatives of the PefCD (RscAB) proteins are the components of the MDR transporters, SatAB of *S. suis* [37], PatAB from *S. pneumonia* [38], and LmrCD (aka YdaG and YdbA) from *L. lactis* [39]. For simplicity we continue in this manuscript to refer to the GAS exporter as the PefCD system. Further *in-silico* study performed on genomes of streptococci identified putative homologs of the *pefRCD* gene cluster in *S. equi*, *S. uberis*, *S. suis* and *S. iniae* [40–44]. A high degree of conservation in genetic organization and sequence exhibiting 54 % amino acid similarity for the PefR, 60 % for the PefC, and 64 % for the PefD protein among all the candidates tested by ClastalW pairwise alignment tool [45]. (Fig. 1a).

### The GAS pefRCD genes entail a heme-induced operon

The *pefRCD* locus is preceded with a 300 bp intergenic region and consists of three overlapping ORFs oriented in the same direction on the direct strand in GAS genome. This genetic organization and the co-regulation by heme indicate that the *pef* locus is an operon. We used RT-PCR analysis to test this suggestion (Fig. 1); RNA was extracted from NZ131 strain after heme exposure and cDNA was synthesized with a primer specific to the complementary strands of *pefD*. The genes encoded by the produced cDNA were subsequently detected by PCR analysis with gene specific primers (see Materials and Methods). These experiments demonstrated that the cDNA produced with the *pefD* primer codes for *pefD*, *pefC* as well as *pefR* (Fig. 1b, lanes 2, 5, and 8), establishing that the *pefRCD* cluster expresses a polycistronic mRNA. No products were observed when total RNA (without RT reaction) was used as a template.

### Construction of a polar mutation in pefC

**Fig. 2:**
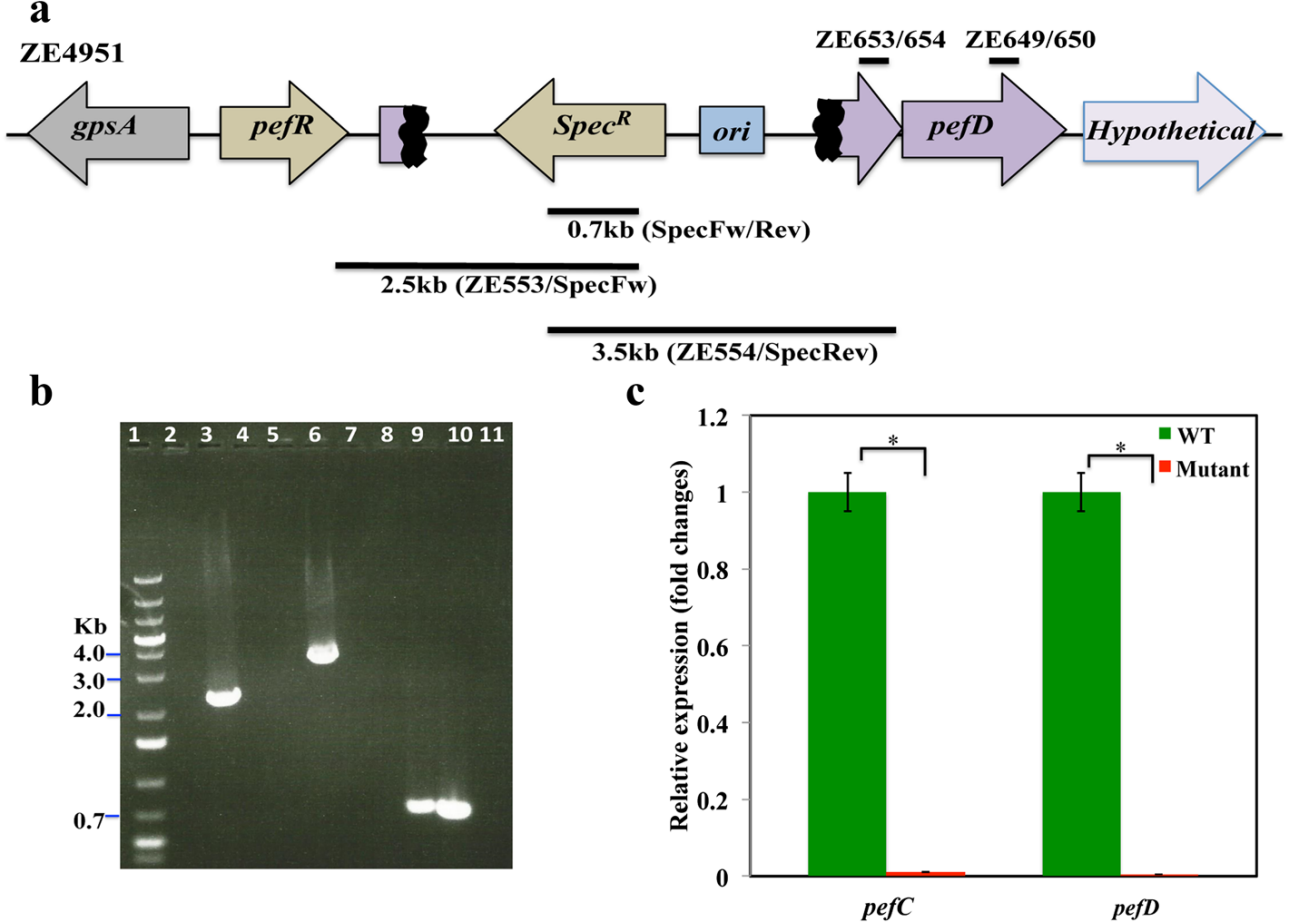
The construction of a polar mutation in *pefC*. **a** Schematic representation of the *pefC::pMZ1* mutation in ZE4951 strain. The lines below the locus diagram denote the DNA fragments amplified by the analysis described in section B. The lines above the diagram denote the products the Q-PCR described in section C. *Spec* ^*R*^ signifies the spectinomycin resistance *aad9* gene and *ori* represents pMZ1 origin of replication*.* The black regions in the *pefC* gene denote the internal fragment that was cloned into pMZ1 and is thus duplicated in the mutant chromosome. **b** PCR analysis of the *pefRCD* locus in the ZE4951 mutant. DNA was amplified by PCR and fractionated on 0.8 % agarose gel. The DNA ladder is shown in lane 1. For the analysis of the left chromosome/plasmid junction the reactions were done with the ZE553/SpecFw primer set and genomic DNA of NZ131 (lane 2), ZE4951 (lane 3), or no DNA (lane 4). For the right plasmid/chromosome junction, the reactions consist of the ZE554/SpecRev primer set and genomic DNA of NZ131 (lane 5), ZE4951 (lane 6), or no DNA (lane 7). For the *spec* ^*R*^ cassette, the reactions were preformed with the SpecFW/SpecRev primer set and genomic DNA of the NZ131 (lane 8), ZE4951 (lane 9), pMZ1 (lane 10, positive control), or no DNA (lane 11). **c** Relative expression of the *pefC* and *pefD* genes in ZE4951 and NZ131 strains. Total RNA was extracted and the relative expression of the *pefC* and *pefD* genes was evaluated by Q-PCR. The relative expression of the *pefC* and *pefD* genes was normalized to *rpsL* transcript levels. The asterisk (*) indicates P value of statistical significance (*P* <0.05) tested using student *t*-test (assuming equal variance) at 0.05 levels of significance

In order to gain insights into the role of the *pefCD* genes in GAS physiology, we created a polar mutation in *pefC* by Campbell insertion (ZE4951 strain) in the background of the wild type strain, NZ131 (Table 1 and Fig. 2a). The formation of the *pefC::pMZ1* mutation was confirmed by PCR analysis. This analysis established the presence of the *spec*^*R*^ cassette in ZE4951 chromosome (the 0.7 kb band in lane 9, Fig. 2b). In addition, these experiments verified the formation of the two expected plasmid/chromosome junctions; specifically, the region spanning *spec*^*R*^ and downstream up to the *pefR* gene (the 2.5 kb band in lane 3, Fig. 2b) and the region covering *spec*^*R*^ and upstream up to the *pefC* portion that is outside of the fragment that was cloned into pMZ1 (the 3.5 kb band in lane 6, Fig. 2b). The same PCR reactions did not produce any product when performed with the chromosomal DNA of the parental strain, NZ131 (lanes 4 and 7 in Fig. 2b). Together, this analysis confirmed that pMZ1 integrated into the correct site in the chromosome forming ZE4951 strain.

**Table 1:** Lists of bacterial strains and plasmids used in this study

Strain name	Characteristics	References
*S. pyogenes*		
NZ131	M49 serotype isolated from acute post-glomerulonephritis infection	[57]
ZE4951	NZ131 derivative with *pefC::pMZ1* mutation	This study
ZE4951/pANKITA5b	ZEM4951 strain complemented with *pefRCD* locus expressed from plasmid pANKITA5b	This study
ZE4951/pKSM201	ZEM4951 strain harboring the pKSM201 vector	This study
*E. coli*		
DH5α	*hsdR17 ecA gyrA endA1 relA1*	[58]
Plasmids		
pMZ1	pUC-Spec derivative containing pefC internal fragment and the spec resistance gene *aad9*	This study
pKSM201	Shuttle vector containing the kanamycin resistance gene *aphA3*	[56]
pANKITA5b	pKSM201 derivative carrying the *pefRCD* genes	This study

To evaluate the effect of the *pefC* mutation on gene expression, we preformed Q-PCR analysis with total RNA extracted from the wild type (NZ131) and the mutant (ZE4951) strains after exposure to heme (Fig. 2). The transcript level of *pefC* (after the insertion site) was reduced to 0.9 % and that of *pefD* to 0.3 % of the level observed in the parental strain (Fig. 2c). Therefore, inactivation of *pefC* in ZE4951 effectively ensued in the loss of the *pefCD* transporter.

### Inactivation of the *pefCD* system *resulted in a growth phenotype and heme hypersensitivity*

**Fig. 3:**
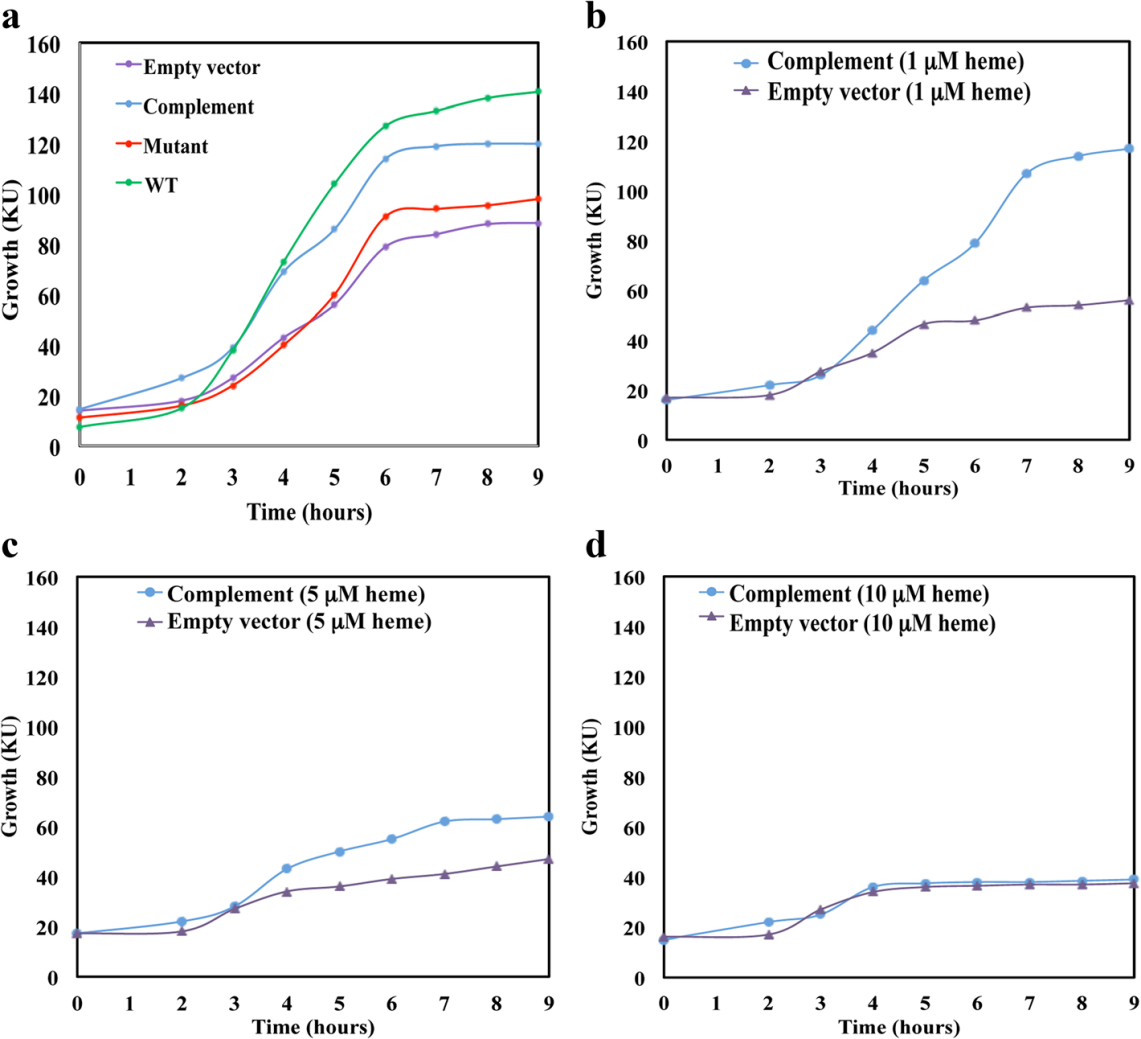
Insertion inactivation of *pefC* in GAS leads to impaired growth and heme hypersensitivity. **a** Growth of the NZ131 (WT), ZE4951 (Mutant), ZE4951/pANKITA5b (Complement), and ZE4951/pKSM201 (Empty vector) strains in THYB. Fresh media were inoculated with GAS cells (OD_600 nm_ = 0.05) and the cultures were grown statically at 37 °C. Cell growth was monitored colorimetrically and expressed in Klett units. **b**-**c**: Growth of ZE4951/pANKITA5b (Complement) and ZE4951/pKSM201 (Empty vector) strains in THYB containing varying heme concentration. The same as with A, only that heme was added to the culture at the early logarithmic phase in final concentration of: **b** 1 μM; **c** 5 μM; and **d** 10 μM. The data are representative of at least two independent experiments

For complementation analysis, we cloned the *pefRCD* operon with its native promoter into a shuttle vector (pKSM201). *E. coli* cells harboring the plasmid carrying the GAS *pefRCD* operon (pANKITA5b) did not exhibit any growth phenotype. However, despite our multiple attempts, we failed to create a sub clone that expresses only the *pefCD* genes (under the *pef* promoter). We therefore used the entire *pefRCD* operon (carried by pANKITA5b) to complement the ZE4951 mutant (Table 1). Analysis of ZE4951 growth in THYB media demonstrated that inactivation of the *pef* system led to a pronounced phenotype; the mutant cells grew slower and reached the stationary phase of growth at a lower cell density in comparison to the isogenic NZ131 wild type strain (Fig. 3a). In addition, cell viability of the mutant strain was significantly reduced during overnight incubation in stationary phase (data not shown). The growth defect of the ZE4951 mutant was reversed in the presence the *pefRCD* genes expressed in trans (pANKITA5b) but not in the presence of an empty shuttle vector (pKSM201, Fig. 3a). This analysis suggests that the *pefCD* genes are required for optimal GAS growth in standard laboratory medium.

When tested for heme sensitivity, the ZE4951 mutant strain exhibited a larger zone of clearance around a heme-submerged disc compared with the wild type strain (Table 2). The mutant sensitivity to heme was restored to the wild type level when complemented in trans with the *pefRCD* genes, but not in the presence of the control plasmid. To further elaborate on the impact of PefCD loss, we compared the growth of the ZE4951 mutant (with empty vector) to that of the complemented strain in THYB containing heme in varying concentration (Fig. 3b-c). We previously determined that the heme MIC of the parental strain NZ131 is 10 μM [15]. In contrast, the growth of the ZE4951 mutant was significantly reduced already in the presence of 1 μM heme (Fig. 3b), and minimal growth was observed with 5 μM heme (Fig. 3c). The complemented strain grew at normal level in 1 μM heme, lower growth was seen in 5 μM heme, and similar to the wild type NZ131 strain, no significant growth was observed with 10 μM heme. These observations establish that the PefCD transporter is required for GAS to thrive in heme containing medium.

**Table 2:** Determination of compound sensitivity by disc diffusion assay

Compounds	Zone of clearance (mm)			
	WT (NZ131)	Mutant (ZE4951)	Complement (ZE4951/pANKITA5b)	Control (ZE4951/pKSM201)
Heme	11.8 ± 1.4	15.8 ± 1.53^a^	9 ± 0.23	13 ± 0.31^b^
Doxorubicin	16	23 ± 1.25^a^	17 ± 0.19	22 ± 1.27^b^
Ethidium Bromide	22.5 ± 0.7	28.8 ± 0.35^a^	24	29 ± 1.4^b^
Hoechst 33342	18	22.8 ± 0.3^a^	19.5 ± 1.8	23.2 ± 1.0^b^
Ampicillin	44.8 ± 1.8	50.7 ± 1.1^a^	45.5 ± 0.8	49.7 ± 1.15^b^
Erythromycin	32 ± 1.0	38.7 ± 0.57^a^	33.3 ± 1.5	40.33 ± 1.52^b^
Norfloxacin	14	14	14.25 ± 0.35	13.75 ± 0.35

The letters ‘^a^’ and ‘^b^’ represent *P* values of statistical significance at 0.05 level of significance calculated using student *t*-test (of equal variance). The statistical significance was evaluated by comparing WT with Mutant & Complement with Control data set

### The PefCD transporter is an MDR system that confers resistance to nuclear stains, antibiotics, and chemotherapeutic agents in addition to heme

The observations described above supported the hypothesis that PefCD consists an efflux system that extrudes heme or heme-related toxic metabolites. While some exporters are selective for a given substrate, many are able to expel a range of structurally unrelated molecules [34, 46]. To test the specificity of the PefCD system we examined the impact of PefCD loss on GAS sensitivity to a variety of structurally unrelated compounds. These included the anthracyclic compound doxorubicin, the antibiotics ampicillin, erythromycin and norfloxacin, as well as the nucleic acid stains ethidium bromide and Hoechst 33342 (Table 2). In comparison to wild type strain, the *pefC* mutant exhibited a larger zone of inhibition around disc containing each of the compounds mentioned above, other than norfloxacin. As with the sensitivity to heme, the drug hypersensitivity phenotype was reversed when the *pefRCD* genes were supplied in trans (Table 2). These data are consistent with the *in silico* analysis that revealed homology between the PefCD proteins and bacterial MDR systems*.*

### Inactivation of the pefCD system is associated with elevated levels of heme-induced damage to cellular constituents

**Fig. 4:**
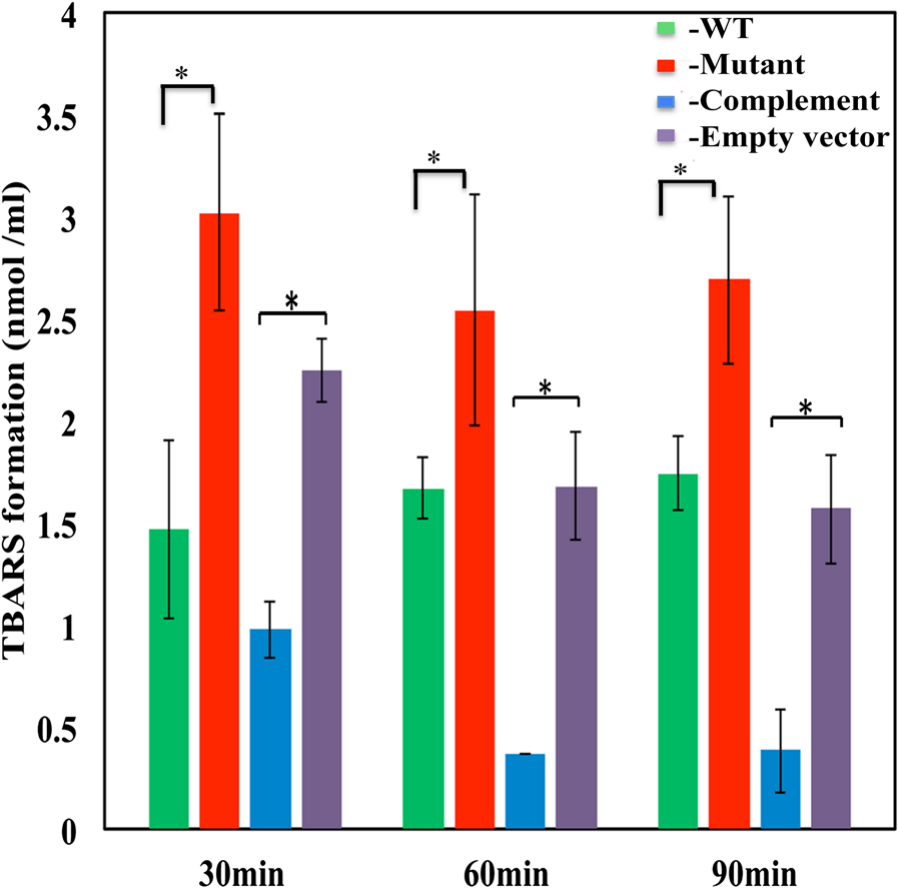
The PefCD transporter protects GAS from heme-mediated lipid oxidation. Cultures of NZ131 (WT), ZE4951 (Mutant), ZE4951/pANKITA5b (Complement), and ZE4951/pKSM201 (Empty vector) strains were treated with 1 μM heme during the mid logarithmic phase of growth (60–70 Klett units). Culture samples were then collected at 30, 60, and 90 min post-heme exposure and allowed to react with TBA. The sample absorption at 532 nm was determined, and the formation of TBA-reactive-substances (TBARS) was calculated using the standard curve shown in A. All samples were standardized with respect to cell number. The data are derived from two independent experiments, each done in triplicates. The asterisk (*) denotes that the observed *P* value is statistically significant (*P* < 0.05) calculated using student *t*-test (equal variance) at 0.05 levels of significance

In order to uncover why the loss of the *pefCD* genes leads to reduced heme tolerance in GAS, we investigated the impact of heme on GAS cellular components in the wild type and mutant strains. In a recent study we demonstrated that exposure to low heme concentration was sufficient to damage the lipids in GAS envelope [15]. Oxidized lipids react with a thiobarbituric acid (TBA) reagent to form adducts (named TBARS) that can be monitored by spectroscopic methods and quantify with a standard curve [15, 47]. We compared the time course of TBARS formation between the wild type and mutant strain after the addition of 1 μM heme into the culture (Fig. 4). Analysis of samples collected 30 min post exposure revealed that the ZE4951 mutant exhibited higher level of lipid damage than the wild type strain (3 versus 1.5 nmol/ml TBRAS in the mutant and wild type strain respectively). Complementation of the *pef*C mutation with the *pefRCD* genes resulted in a significantly lower level of TBARS formation, in comparison to mutant cells harboring the negative control plasmid (0.97 and 2.49 nmol/ml respectively). Interestingly, the TBARS levels in the complemented mutant strain were reduced over time (from 0.98 to 0.38 nmol/ml in the 30 and 90 min samples respectively), while they remained approximately the same in the wild type, mutant, and the mutant cells harboring the control plasmid. Together these observations imply that the PefCD system defends GAS from heme-induced lipid damage and suggest that the expression of *pefRCD* genes under our experimental conditions may be limiting in the wild type strain.

**Fig. 5:**
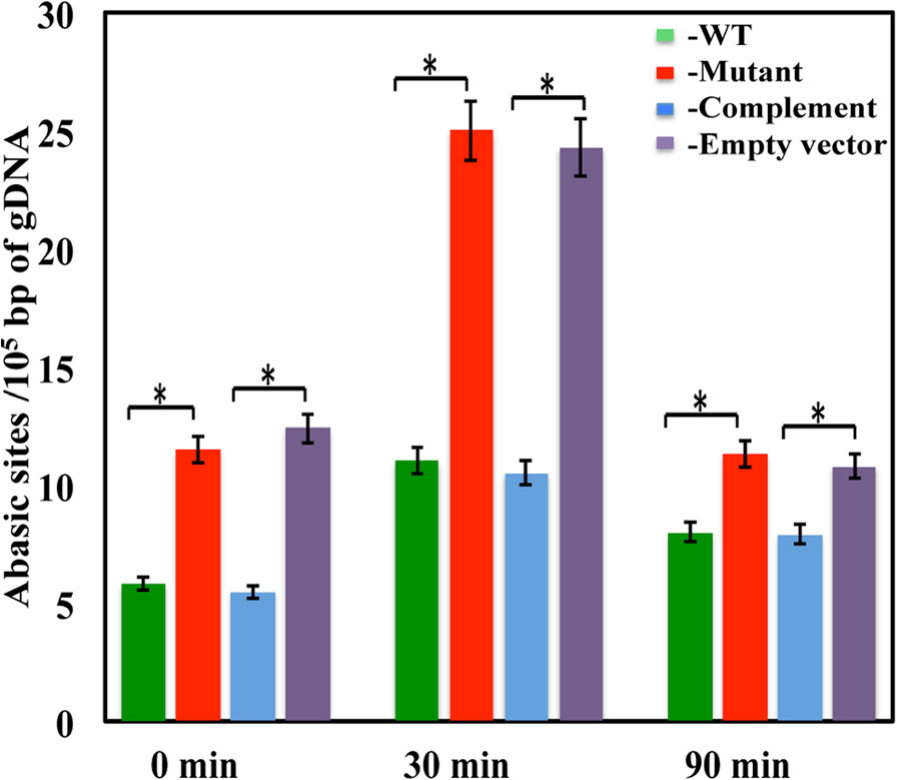
The PefCD transporter protects GAS chromosome from heme-mediated damage. Cultures of NZ131 (WT), ZE4951 (Mutant), ZE4951/pANKITA5b (Complement), and ZE45/pKSM201 (Empty vector) strains were treated with 5 μM heme during the mid logarithmic phase of growth (60–70 Klett units). Genomic DNA was extracted from samples collected at 0, 30 and 90 min post exposure was allowed to react with ARP-biotin and analyzed. The sample absorption at 650 nm was determined and AP site formation was calculated using the standard curve shown in A. All samples were standardized with respect to cell number. The data are derived from two independent experiments each done in triplicates. The asterisk (*) denotes that the observed P value is statistically significant (*P* < 0.05) and is calculated using student *t*-test (equal variance) at 0.05 levels of significance

Studies performed with eukaryotic systems showed that heme could harm nucleic acids [48]. We asked if environmental heme can damage GAS genome and if the *pef* system offers protection from this harm. The repair of chemically altered bases (due to oxidation, deamination or alkylation) in the DNA is often mediated by repair mechanisms that involve the formation apurinic/apyrimidinic (AP) sites. Therefore, the amount of AP sites serves as a good indicator for DNA damage and repair. To quantitate the formation of AP sites in GAS chromosome, we used a reagent (ARP) that reacts specifically with the aldehyde group in the open ring form of the AP sites [49]. Chromosomal DNA was extracted from culture samples that were harvested at different time points and was allowed to react with ARP reagent. The formation of AP sites was detected by ELISA and quantified using a standard curve (Fig. 5). Analysis of chromosomal DNA from culture samples of the wild type strain collected 30 min after the addition of heme revealed about 90 % increase in the number of AP sites compared to the background level (11 and 5.8 AP sites per 10^5^ bp respectively). The AP sites level was reduced over time, but remained 40 % above the background (8 AP sites per 10^5^ bp) in the samples collected 90 min after treatment. Interestingly, in *pefC* mutant the background level of AP sites was about twice as high compared with the wild type strain (11.5 AP sites per 10^5^ bp). Heme exposures resulted in a transient 117 % increase in the number of AP sites, which was reduced to background level within 60 min (25 and 11.5 AP sites per 10^5^ bp in the 30 and 90 min respectively). The high AP levels observed in the mutant strain were complemented to those observed in the wild type strain by the *pefRCD* genes expressed from a plasmid in all samples. The levels of AP sites in the mutant strain carrying the empty vector were comparable to that of the mutant strain alone. These experiments indicate that environmental heme led to DNA damage in GAS and that the *pefCD* genes offered protection from DNA damage during growth in laboratory medium as well as in medium containing externally added heme. In addition, our data highlights the presence of DNA repair mechanisms in GAS.

### The PefCD system prevents cellular heme accumulation

**Fig. 6:**
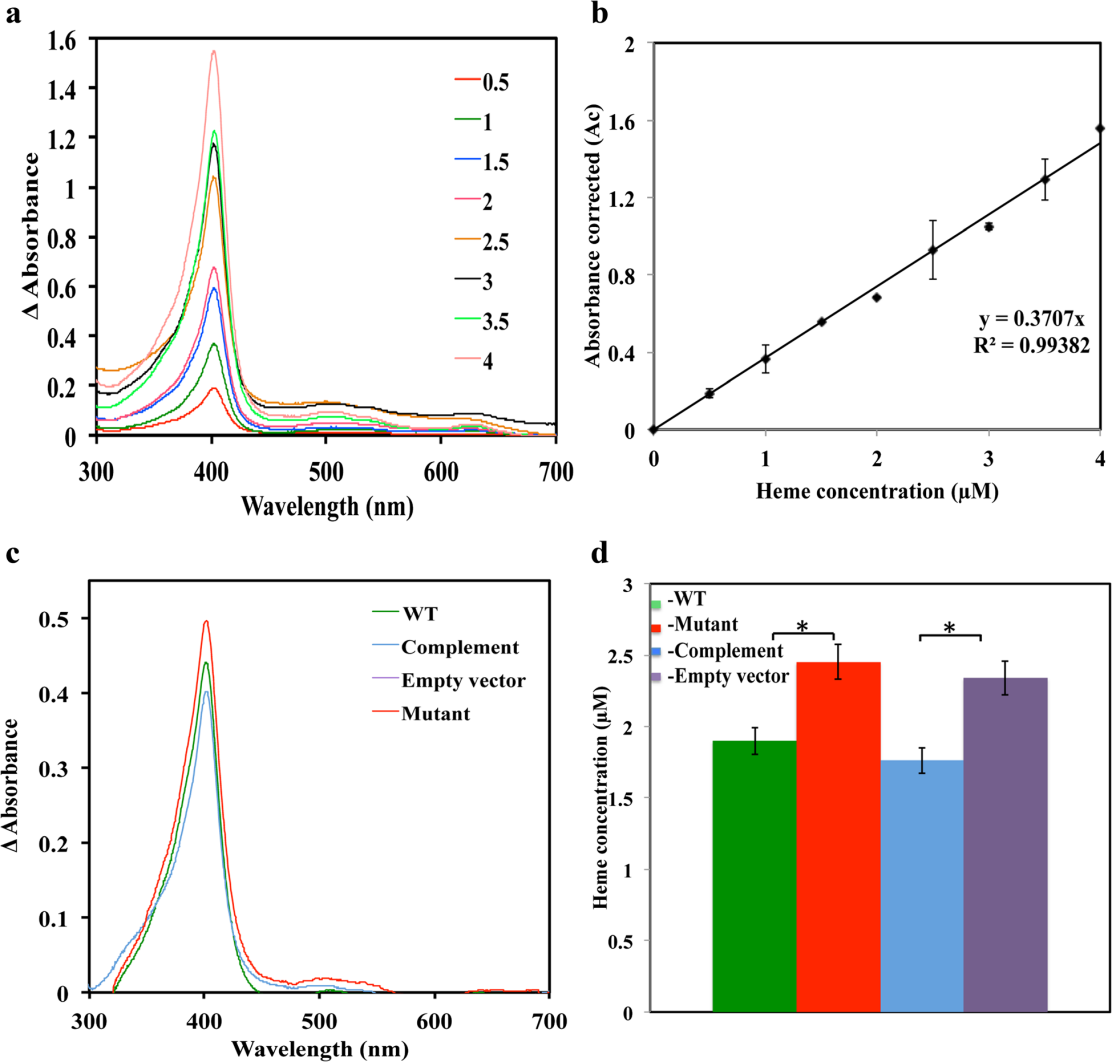
Inactivation of the *pefCD* transporter leads to cellular accumulation of heme in cells grown in the presence of heme. **a** UV-visible spectra across wavelengths (250–700 nm) were recorded for organic fractions recovered after acidified chloroform extraction performed on a range of hemin chloride standards. **b** The observed absorbance at 388, 450, and 330 nms from UV scans (of organic fractions) were plugged into *A*_c_ = 2*A*_388_ − (*A*_450_ + *A*_330_) equation. The A_c_ values for standards extracted using chloroform for a range of hemin concentrations (0–4 μM, with 0.5 μM increments) were plotted against its hemin concentrations to generate a standard plot. The line equation of a standard plot was used to extrapolate hemin concentrations in the experimental samples. Cultures of NZ131 (WT), ZE4951 (Mutant), ZE4951/pANKITA5b (Complement), and ZE4951/pKSM201 (Empty vector) strains were treated with 3 μM heme during the mid logarithmic phase of growth (60–70 Klett units). Cells were harvested, washed, and were subjected to chloroform extraction. **c** UV-visible spectra across different wavelengths (250–700 nm) of the collected organic phases from tests samples were recorded. **d** Heme concentration in the test samples. The concentrations of heme in the test samples were calculated using the standard curve shown in B. The data are derived from two independent experiments each done in triplicates. The asterisk (*) denotes that the observed *P* value is statistically significant (*P* < 0.05) calculated using student *t*-test (equal variance) at 0.05 levels of significance

One interpretation of the experiments described above is that following the addition of heme to the culture medium GAS cells absorb it in surplus amounts; in the absence of a functional PefCD system, the bacterium accumulates heme or heme-related metabolites that damage the cell constituents. To directly test this hypothesis, we examined the cellular heme content by the acidified chloroform extraction method [50] described in the material and methods (Fig. 6). Culture samples of equal cell density were collected 90 min after the addition of 3 μM heme to the medium and the cells were washed extensively to remove surface bound heme prior to heme extraction. The presence of heme in the organic phase of the cell extracts was examined by spectroscopic analysis and the concentration of the extracted heme from each sample was extrapolated from a standard curve (Fig. 6b and d). We extracted in these experiments about 30 % more heme from the *pefC* mutant in comparison to the wild type strain. The heme concentration obtained from cells of the *pefC* mutant complemented with the *pefRCD* genes, was 7 % lower than that extracted from the wild type cells. On the other hand, the presence of the empty vector did not have a significant impact on the heme concentration in the extract from the *pefC* mutant. Therefore, the PefC accumulates more heme then the wild type strain. This observation suggests that the PefCD system expels surplus heme from GAS cells.

## Discussion

Heme is an important nutrient for the β-hemolytic GAS, which uses it in order to obtain iron within the host environment [8]. Despite its nutritional value, heme is a harmful molecule that is damaging to GAS envelope even in low concentrations [15]. In the current study, we investigated how GAS manages the toxic challenges imposed by environmental heme. We recently identified a 3-gene cluster that was induced by heme and named it after the *pefRCD* system in GBS [15, 32]. Following up on our initial observations, we demonstrated that *pefRCD* genes entail an operon and carried out a functional characterization of the *pefCD* genes in the GAS strain, NZ131. We established that p*efCD* encode an MDR exporter that plays an important role in heme tolerance. The PefCD proteins defend GAS membrane and chromosome from the toxic effects of heme stress and prevent heme accumulation in the bacterial cells. This is the first heme tolerance pathway to be described in GAS.

To examine the role of the *pefCD* genes in GAS, we created a polar mutation in *pefC*, which we then supplemented with the *pefRCD* genes expressed from a plasmid (since we could not clone *pefCD* under the native *pef* promoter in the absence of *pefR*). The observed heme related phenotypes of the *pefC* mutant were effectively complemented. These observations indicate that under our experimental conditions, the PefCD proteins are expressed *in trans* at a level that is sufficient to mitigate the negative impact of heme. Our failed attempts to clone *pefCD* in the absence of *pefR*, raises the possibility that the constitutive expression of the *pefCD* genes is detrimental to *E. coli*. Toxicity of membrane proteins when overexpressed or of proteins expressed in heterologous hosts is not uncommon. A similar problem was reported for the related SatAB system of *S. suis* [37].

Examining the phenotype of the *pefC* mutant and the complemented strain showed that the loss of the PefCD system led to increased sensitivity to heme and to five structurally unrelated drugs (Table 2). This observation is consistent with the bioinformatics analysis that revealed significant similarity among PefCD proteins and the confirmed MDR transporters SatAB, PatAB, and LmrCD from *S. suis*, *S. pneumoniae* and *L. lactis* respectively [37, 39, 51]. Together, these findings indicate that the PefCD proteins consist of a MDR transporter that expels antimicrobial compounds as well as heme or heme related metabolites. Our additional findings demonstrated that GAS accumulates heme in the absence of a functional PefCD transporter (Fig. 6), strongly implicating heme as one the substrates of the *pefCD* system. The *pefC* mutant exhibited growth attenuation in THYB (Fig. 3a). THYB contains infusions of brain and heart tissues. Therefore, the mutant growth phenotype might have resulted from increased sensitivity to the heme that is already present in the medium. Alternatively or in addition, PefCD may help in the clearance of other toxic molecules that are generated during growth. Interestingly, a previous study performed in the JRS4 strain revealed that the *pefC* (*rscA*) gene is induced at low pH and by heat stress and that a *pefC* mutant could not grow at 40 °C. The growth phenotype at a high temperature raised the possibility that the *pefCD* proteins export molecules that harm the membrane structure and fluidity [36]. Heme could be such a molecule, since it is known to penetrate membranes and undermine the membrane permeability [30, 52]. Alternatively, other compounds exported by PefCD may be responsible for this phenotype.

We previously reported that the addition of 4 μM heme to the culture medium produced oxidative damage in the envelope of the invasive MGAS5005 strain [15]. In this work, we made similar observations with the nephritogenic NZ131, and showed that exposure to 1 μM heme led to noticeable levels of lipid peroxidation (Fig. 4). Moreover, we showed for the first time that externally added heme is also damaging to GAS chromosome (Fig. 5). Together, these observations highlight the destructive impact of environmental heme on GAS cellular components even at relatively low concentrations. A number of observations demonstrate the importance of the PefCD system in heme detoxification and suggest a positive correlation between the expression of *pefCD* system and detoxification capacity: 1) The *pefC* mutant experienced more lipid and DNA damage comparing with the wild type strain after heme treatment (Figs. 4 and 5). 2) The level of lipid peroxidation in the wild type strain remained constant for 30–90 min post treatment, while it was reduced over time in the complemented strain (Fig. 4). 3) More AP sites were detected in the chromosome of the *pefC* mutant comparing to the wild type strain even in the absence of externally added heme. 4) Heme begins to accumulate in the mutant cells after its addition to the growth medium (Fig. 6d). The *pef* regulon in GBS is induced by levels of heme that are lower than those required to fully activate the putative HrtAB transporter; it was therefore suggested that the Pef exporters allow for adjustments in small variations in heme and PPIX concentrations under different physiological conditions while the Hrt exporter may be needed to defend GBS against higher heme concentrations [32]. Here, we established that the PefCD system in GAS is responsive to low levels of environmental heme (1–5 μM) and participate in the detoxification process. To the best of our knowledge, a functional HrtAB-like transporter was not described in GAS up to date. Nevertheless, GAS genome contains putative ABC transporters that belong to the same transporter class as HrtAB. It is possible, that like with GBS, these or other transporters are recruited in GAS at higher heme concentrations in addition to PefCD.

GAS is a versatile pathogen that exhibits wide tissue tropism and adaptability that enables it to inhibit not only the epithelia of the skin and mucous membranes, but also to flourish within soft tissues, the lymphatic system, and the blood [2]. Therefore, depending on the nature and stages of infection, GAS is expected to confront a wide spectrum of heme concentrations, ranging from low amounts (e.g., during simple skin or mucosal infection) to very high concentrations (such as during invasive infections associated with significant erythrocyte lysis and tissue destruction). Furthermore, heme tolerance varies among GAS isolates with highly invasive strains such as MGAS5005 showing higher resistance (Heme MIC = 50 μM) than strains that are typically associated with local infections such as NZ131 (MIC = 10 μM) [15]. We propose that the PefRCD represent a fundamental heme defense pathway that is shared by all GAS strains, safeguarding the organism against environmental heme. Highly invasive strains may carry additional pathway (s) that might confer increased resistance.

**Fig. 7:**
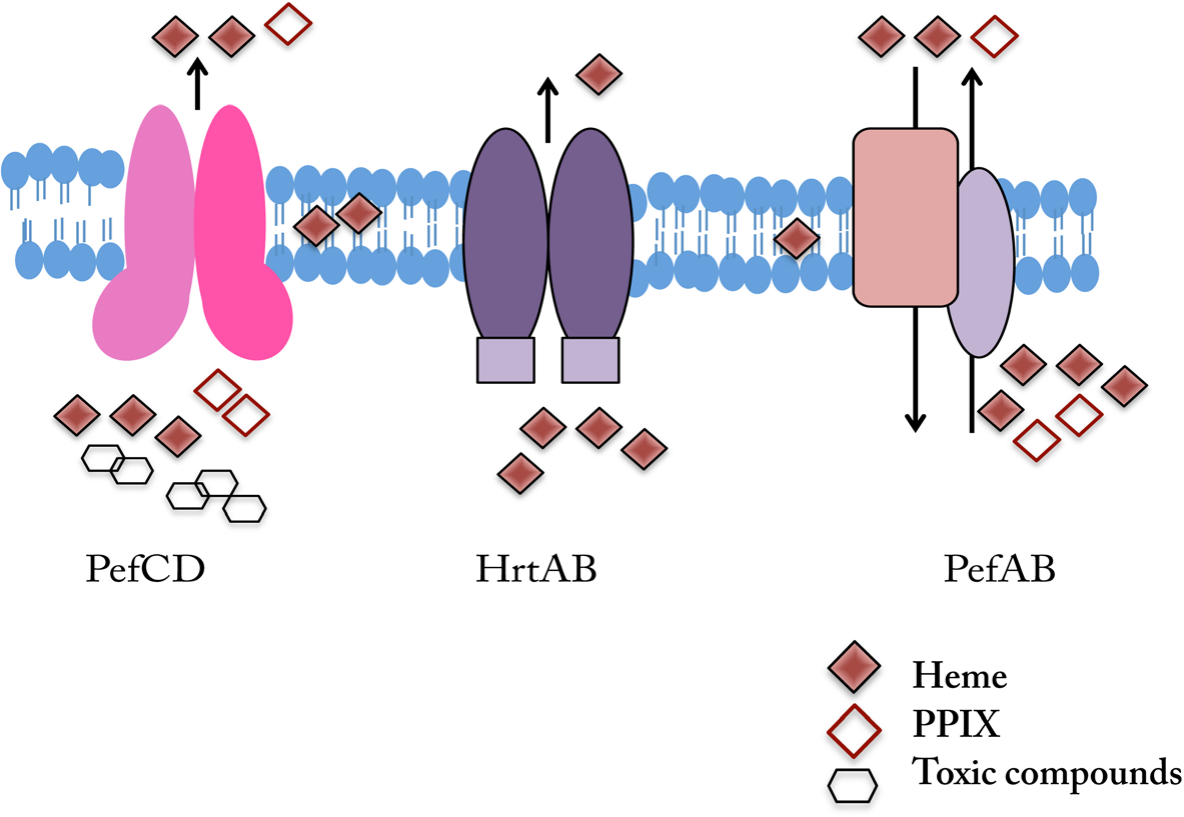
A schematic depiction of heme-tolerance transporters described in streptococci. Putative homologs of the HrtAB (Class-3 ABC-type transporter) were identified in GBS, and were shown to be induced by heme. The PefAB and PefCD are heme and PPIX efflux machineries used by GBS. PefAB is related to the drug/proton antiporter family, while PefCD consists of Class-1 ABC type transporter. Our data show that GAS employs PefCD to efflux heme and various antibacterial compounds

The role of active export in the management of heme (and other porphyrins) levels and toxicity in bacterial systems is not fully appreciated and only few systems have been described up to date. Two systems were identified in Gram-negative bacteria (MacAB/TolC and MtrCDE for *E. coli* and *N. gonorrhoeae,* respectively) [22, 25, 53]. Both exporters consist of cytoplasmic transporters that connect via a membrane fusion protein to an outer membrane channel/tunnel protein. Both cytoplasmic transporters are MDR systems that export a number of compounds in addition to heme and/or porphyrins. However, MacAB is an ATP-type transporter while MtrC belongs to the Resistance/Nodulation/Division (RND) family of transporters, which uses the proton motive force for energy-dependent export. In Gram-positive bacteria, four types of energy dependent exporters used for heme and/or PPIX detoxification were described (Fig. 7 portrays the heme transporters described in streptococci). Intriguingly, HrtAB, which was first identified in *S. aureus* [27], belongs to the MacAB family of ABC-type efflux carriers. In this family of ABC transporters, the membrane permease (IM) and the ATPase (ABC) domains are encoded by separate polypeptides (Class 3) [34]. HtrAB mediates heme tolerance in a number of Gram-positive bacteria. It was shown to pump heme directly out of the membrane compartment in *L. lactis* [30] and it is likely that it functions in a similar manner in the other Gram-positive species. In *L. monocytogensis*, a P-Type ATPase, that displays homology to bacterial heavy-metal transporting ATPases, confers resistance to heme and hemoglobin [33]. The two other types of transporters were described first in GBS: PefAB (antiporter) belongs to the MFS family that relies on the proton motive force and PefCD, which is a Class I, ABC-type transporters [32]. In this study, we showed that the GAS PefCD system contributes to tolerance against both heme and a wide range of antimicrobial compounds. The involvement of the other transporters from Gram-positive bacteria in resistance to compounds other than porphyrins was not examined.

## Methods

### Bacterial strains and growth conditions

Plasmids and strains used in this study are listed in Table 1 and the primers are described in Table 3. *E. coli* cells were used for cloning and propagation of plasmids. *E. coli* was grown aerobically in Luria-Bertani medium (BD) at 37 °C (pH 7.0). GAS was grown under static conditions in Falcon tubes or nephelometer flasks in Todd-Hewitt broth (TH broth, Difco laboratories) with 0.2 % (weight/volume) yeast extract (BD). GAS ZE4951 mutant and the complemented strains (carrying pANKITA5b or pKSM201) were grown with Spectinomycin or with Spectinomycin and kanamycin, respectively. Growth was measured using a Klett-Summerson colorimeter (A filter) and/or by viable counting. In some cases, hemin chloride (Sigma) prepared in DMSO (0.035 % final concentration, Fisher BioReagents) was added to the cultures. Spectinomycin was used at 100 μg/ml for both *E. coli* and GAS, and kanamycin at 70 μg/ml for *E. coli* and 300 μg/ml for GAS.

**Table 3:** Lists of primers used in this study

Primers name (Target)	Restriction site (Use)	Sequence (5’–3’)	Source
ZE565 (*pefC*)	EcoR1	GGGGAATTCTTATGGTGGGTCATTGTTG	This study
ZE566 (*pefC*)	EcoR1	AATGAATTCTAAGCGAGGGATGAGCTGTG	This study
ZE634 (*pefRCD*)	SpeI	AAGGACTAGTGGTCTTGGCTAATAAGGCG	This study
ZE635 (*pefRCD*)	SpeI	AGGGACTAGTTTGGGATTCATGTTAGCGAG	This study
ZE553 (*pefR*)	NA	TGAGGAGACAACGATGCTTAAAGAC	This study
ZE554 (*pefC*)	ClaI	AAAAAATCGATCAGGGTTGTTTGACTTAG	This study
ZE458 (*pefR*)	NA	CACAAGTGATAGGTGATTTACGTG	This study
ZE562 (*pefR*)	NA	CCTTGAGGACCTGCTAGATGCTCTAC	This study
ZE649 (*pefD*)	NA	GGAGAATTGCAGCTGGCC	This study
ZE650 (*pefD*)	NA	AGCTGGTGAACGCTGGTGC	This study
ZE653 (*pefC*)	NA	GCGTTCAAGAAGGCCTTAAGTC	This study
ZE654 (*pefC*)	NA	CCACCAACTCTGCGTGTGTTC	This study
ZE581 (*rpsL*)	NA	CAGATTCACCAGCTTTGAAC	[15]
ZE582 (*rpsL*)	NA	CAACACGAGTAGCAACG	[15]
SpecFw	NA	GTGAGGAGGATATATTTGAATACATACGAA	This study
SpecRev	NA	GTCCATTCAATATTCTCTCCAAGATAACTA	This study

### Nucleic acid methods

The nucleic acid methods were performed with kits and enzymes according to the manufacture’s instruction, unless specified otherwise*. DNA manipulations:* Chromosomal and plasmid DNA extraction and DNA manipulations were done according to standard protocols and as previously described [54]. Plasmid DNA was isolated from *E. coli* using the Wizard Plus SV mini-prep system (Promega). DNA was subjected to restriction digestion (SpeI-HF® and or EcoRI of NEBL), and fragments were gel purified from agarose using S.N.A.P. UV-free gel purification kit (Invitrogen). The digested and gel purified fragments were ligated using T4 DNA ligase (Roche) to generate a recombinant construct. *RT-PCR:* RT-PCR was used to test if *pefRCD* genes are co-expressed. Total RNA was extracted (90 min after treatment with 3 μM heme) using the RiboPure™ Bacterial RNA isolation kit (Ambion™) and DNA contamination was removed with DNase I (Ambion™). PCR performed with 1 μg RNA as a template was used to confirm the absence of DNA contaminations. cDNA was synthesized from 0.8 μg RNA by reverse transcriptase (ProtoScript® II, NEB) using the *pefD* antisense primer, ZE650 (5 μM). The presence of *pefR*, *pefC* and *pefD* genes in the cDNA was tested by PCR with the following primer sets: ZE458/ZE562, ZE651/ZE652, and ZE649/ZE650 (Table 3). *Real-Time RT-PCR (Q-PCR):* Q-PCR was used to compare the expression of the *pefC* and *pefD* genes between the wild type and the mutant strains. Briefly, 25 ng of DNase-I digested, ultra-pure total RNA was added to 20 μl Power SYBR® green master mix (AB), containing enzyme and 200 nM of gene specific primers (ZE653/ZE654 for *pefC* and ZE649/ZE650 for *pefD*). The reactions were preformed following the One Step RNA to C_T_ protocol, using 7500 Fast Real-Time PCR instrument (AB). Data acquisition and analysis involved relative quantification with comparative ΔΔC_T_ process. The relative expression of the *pefC* and *pefD* genes was normalized to *rpsL* transcript levels, which was used as an endogenous control.

### Construction of plasmids, GAS mutants and complementation strains

The *pefC::pMZ1* mutant, ZE4951, was constructed in NZ131 background by Campball insertion using plasmid pMZ1. Briefly, an internal 696 bp fragment from the *pefC* gene was amplified from NZ131 chromosome using ZE565 and ZE566 primers and ligated into the EcoRI site in pUC-Spec, which carries the spectinomycin resistance gene, *aad9* [55]. The resulting plasmid, pMZ1, was introduced into NZ131 strain and spectinomycin resistant colonies were selected. The structure of the chromosomal *pefC::pMZ1* mutation was verified by PCR and sequence analysis. For complementation analysis, a 4336 bp fragment spanning the p*efRCD* operon including the promoter region was amplified from NZ131 chromosome with ZE634 and ZE635 primers and ligated into the SpeI site of the shuttle vector, pKSM201 [56], which carries the kanamycin resistance gene *aphA3*, generating pANKITA5b. The plasmids pKSM201 (empty vector) and pANKITA5b were introduced into ZE4951 strain by selection for spectinomycin and kanamycin resistance.

### Disc diffusion assay

GAS sensitivity was tested using the Disc Diffusion method as previously described [15]. Briefly, sterile 8.0 mm Whatman filter paper discs (with 1.2 mm width) were submerged in doxorubicin (1.6 mM), heme (10 mM), ampicillin (10 mM), erythromycin (10 mM), Hoechst 33342 (20 mM), ethidium bromide (24 mM) solutions or Norfloxacin (10 μg, ready to use disc, OXOID), dried and impregnated onto THYA that was plated with 0.1 ml (OD_600nm_ = 0.1) of overnight GAS culture. The plates were incubated at 37 °C for 17 h before the zone of clearance around the discs was measured.

### Determination of lipid peroxidation using TBARS assay

Fresh THYB was inoculated with GAS cells collected from plates: the inocula consisted of cells grown overnight at 37 °C on THYA and suspended in saline. Bacterial suspensions at OD_600_ = 0.5 were diluted 1:100 into fresh THYB, incubated at 37 °C, and growth was monitored. Heme (1 μM) was added at the logarithmic phase of growth (~60–70 Klett units) and samples (standardized according to cell density) were harvested by centrifugation (5000 × g, 20 min at 4 °C) at 30, 60, and 90 min post exposure. The cell pellets were washed and samples were prepared and analyzed for lipid peroxidation using OXI-TEK TBARS (Thiobarbituric Acid Reactive Substances) assay kit (ZeptoMatrix Corporation) as previously described [15]. Sample absorbance at 532 nm was recorded using the DU 730 Life Science UV/vis spectrophotometer. The A_532 nm_ obtained for the experimental samples were compared to a standard curve generated using the malondialdehyde (MDA) reagent supplied with the kit.

### DNA damage detection

Fresh THYB medium was inoculated with GAS cells collected from THYA plates as described above. The cultures were treated with 5 μM heme at the logarithmic phase of growth (~60–70 Klett units) and samples (standardized according to cell density) were harvested at 0, 30 and 90 min post exposure. Genomic DNA was extracted from the cell pellet using the ArchivePure DNA extraction kit (5 PRIME). DNA purity and concentration were determined and 0.5 μg of DNA samples were used to quantify the formation of apurinic/apyrimidinic (AP) sites using the DNA damage detection kit (PromoKine) per the manufacture instructions. Briefly, DNA (500 ng) was incubated with aldehyde reactive probe containing biotin tag (ARP) solution for 1 h at 37 °C. Glycogen and TE buffer were added to the solution, the DNA was allowed to precipitate at −20 °C for 10 min and then collected by centrifugation at 13,000 rpm for 10 min at 4 °C. The pellet of the biotin-tagged DNA was washed with 70 % ethanol, air-dried, resuspended in 1 ml of TE buffer. The DNA samples (60 μl) were added to 96 well ELISA plate and incubated overnight at room temperature with DNA binding solution. The signal was developed after incubation with horseradish peroxidase (HRP)-streptavidin solution and developer. The signal intensity was quantified spectroscopically at 650 nm. A standard plot depicting the absorbance at 650 nm as a function of the number of standard AP sites of standard solution (0, 8, 16, 24, 32, and 40 ARP sites/10^5^ bp solution) was used to determine the number of AP sites/10^5^ bp DNA fragment in experimental samples. As a positive control for AP site formation in GAS cells, we used cells exposed to 10 mM H_2_O_2_ solution for 1 h at 37 °C followed by genomic DNA extraction, labeling, and detection similar to standards and experimental samples (data not shown).

### Determination of intracellular hemin content

Fresh THYB medium was inoculated with GAS cells collected from plates as described above. The cultures were treated with 3 μM heme at the logarithmic phase of growth (60–70 Klett units) and samples (standardized according to cell density) were collected 90 min post exposure by centrifugation (8000 × g for 20 min at 4 °C). The cell pellet was washed 5 times (10 ml phosphate buffered saline, pH 7.0 each), resuspended in 2 ml of DMSO, and subjected to sonication (20 % amplitude for 30 s.). Heme content in the cell lysate was determined using the procedure described by Lombard et al. with small modifications [50]. Briefly, 4 ml of experimental samples along with hemin standard solutions (0–5 μM, in DMSO) were subjected to acidified chloroform extraction as follows: addition of 2 ml of 50 mM glycine buffer, pH 2.0 then 0.1 ml of 4 N HCl (pH 2.0), 0.2 ml of 5 M NaCl (pH 2.0) and chloroform (2 ml) followed by vigorous mixing for 10 s (6 times). The reactions were incubated at room temperature for 1 min and the absorbance by the organic phase was then determined. The absorbance at 388, 450 and 330 nm were recorded and fed into the correction equation A_c_ = 2 × A_388_ − (A_450_ + A_330_). The plot of A_c_ corrected for standard heme concentrations was used to extrapolate the hemin concentration in experimental samples.

## Conclusions

We established significant orthology between the GBS PefCD and the system encoded by GAS. Moreover, our *in silico* analysis suggests that the previously described SatAB transporter from *S. suis* is an orthologous system and that a *pefRCD*–like operon is also found in the members of the pyogenic streptococci. The *S. suis*, SatAB, was shown to transfer norfloxacin and ciprofloxacin [37]. In GBS, the PefCD proteins export heme and PPIX [32]. Our work here illustrates that PefCD in GAS exports heme as well as a variety of structurally unrelated compounds. It will be interesting to find out whether the PefCD system is a MDR transporter that has heme as one of its substrates in all of the streptococcal species, or if it has evolved distinct substrate specificities among various species reflecting differences in ecological niches and lifestyle.

### Ethics approval and consent to participant

This study did not involve human subjects, human material, human data, animals or plants.

### Availability of data and materials

Data presented in this study are complete. No supplementary files are attached.

### Open access

This article is distributed under the terms of the Creative Commons Attribution 4.0 International License (http://creativecommons.org/licenses/by/4.0/), which permits unrestricted use, distribution, and reproduction in any medium, provided. You give appropriate credit to the original author(s) and the sources, provide a link to the Creative Commons license, and indicate if changes were made. The Creative CommonsPublic Domain Dedication waiver (http://creativecommons.org/publicdomain/zero/1.0/) applies to the data made available in this article, unless otherwise stated.

## Abbreviations

ABC: ATP binding cassette
AP: apurinic/apyrimidinic
ARP: aldehyde reactive probe
DMSO: dimethyl sulfoxide
GAS: group A streptococcus
GBS: group B streptococcus
MDR: multi-drug resistance
MIC: minimal inhibitory concentration
PCR: polymerase chain reaction
Pef: porphyrin-regulated efflux
TBARS: thiobarbituric acid reactive species
THYB: Todd-Hewitt Yeast extract Broth
